# Examining Workplace Ostracism Experiences in Academia: Understanding How Differences in the Faculty Ranks Influence Inclusive Climates on Campus

**DOI:** 10.3389/fpsyg.2016.00753

**Published:** 2016-05-30

**Authors:** Carla A. Zimmerman, Adrienne R. Carter-Sowell, Xiaohong Xu

**Affiliations:** ^1^Department of Psychology, Texas A&M University, College StationTX, USA; ^2^Department of Psychology and Africana Studies Program, Texas A&M University, College StationTX, USA; ^3^Department of Psychology, Bowling Green State UniversityBowling Green, OH, USA

**Keywords:** workplace ostracism, information exclusion, gender diversity, chilly climate, group status, Faculty of Color

## Abstract

Research on the retention of women in academia has focused on challenges, including a “chilly climate,” devaluation, and incivility. The unique consequences of workplace ostracism – being ignored and excluded by others in an organizational setting – require focus on this experience as another interpersonal challenge for women in academia. The purpose of this study is to examine differences in the faculty experiences and outcomes of workplace ostracism, and to determine if these experiences are affected significantly by the gender composition of an employee’s specific department. Participants were recruited at two time points to complete campus climate surveys that were distributed to faculty at a large, public, research university. We examined the number of reported ostracism experiences (Study 1) and perceived information sharing (Study 2) among male and female university faculty. The findings indicated that female faculty members perceived more workplace ostracism than male faculty members. Analyses of department gender ratios suggested that the proportion of women in the department did not reduce the amount of workplace ostracism experienced by women. No gender differences were found in perceived information sharing. However, we found that Faculty of Color, both men and women, reported more frequent information exclusion than White faculty. These results have important implications for theoretical and practical understandings of workplace demography and suggest that it is necessary to look at subtle, ambiguous forms of discrimination in order to increase retention of faculty from underrepresented groups in academia.

Scrutiny continues over the lack of gender diversity among the faculty ranks at US colleges and universities. Hoover (2015) released a report confronting the disparities in faculty hiring practices. The statistics, across departments on campuses around the country, confirm a systematic pattern for women in the academic labor market. In 2012, women received 46% of all doctoral degrees awarded in the United States, yet female faculty at doctorate-granting institutions accounted for 38% of the professoriate (Integrated Postsecondary Education Data Systems [IPEDS], 2012). In fall 2013, of all full-time, instructional faculty in degree-granting postsecondary institutions, 6% were Black female faculty members, 5% were Hispanic female faculty members, and 7% were Asian/Pacific Islander female faculty members (Catalyst, 2015). Research on the barriers faced by female faculty have focused on interpersonal, micro-level factors, such as implicit bias, microaggressions, and tokenism, along with institutional, macro-level factors, including wage gaps, promotion gaps, and policy lapses.

Our research contributes to the topic of diversity and inclusion in the workplace by replicating and extending research the disproportionate effects of workplace ostracism – being ignored and excluded by others on the job (Carter-Sowell and Zimmerman, 2015). We examine these diverse experiences using two measures of inclusion – first focusing on social interactions, and the second focusing on information sharing – a little studied form of ostracism. Guided by tenets of social identity and social exchange theory, we illustrate group-level differences in experiences of workplace ostracism. Also, we extend the existing literature on women in academia by using organizational records to examine the effects of objective departmental gender composition on reported experiences of workplace ostracism. Lastly, we suggest ways to promote inclusive climates for a gender diverse workforce.

## Workplace Ostracism ≠ Bullying on the Job

Traditionally, universities refer to the students, alumni, staff, and faculty as members of the campus family. Accordingly, individuals strive to be accepted rather than outcast by others in their group. Nonetheless, ostracism is a part of everyday life (Nezlek et al., 2012) and nearly two-thirds of employees reported being ostracized at work (e.g., Fox and Stallworth, 2005; O’Reilly et al., 2014). This experience can take a variety of forms, including being uninformed of information mutually known by others, known as being out-of-the-loop (Jones et al., 2009), being excluded when others are speaking in a language not understood by all who are present, known as linguistic ostracism, (Hitlan et al., 2006b), and/or being given the silent treatment by others (Fox and Stallworth, 2005).

Workplace ostracism can be conceptualized as a workplace stressor, due to its negative effects on targets’ psychological distress, work-related attitudes, and behaviors (Jex et al., 1992). Targets of ostracism experience greater distress than non-targets (Ferris et al., 2008; Sulea et al., 2012; Wu et al., 2012). The attitudinal and behavioral manifestations of strain that result from workplace ostracism are detrimental to the targeted employee, coworkers, and the organization. Job satisfaction and engagement are negatively correlated with workplace ostracism, while job-induced tension is positively related to workplace ostracism (Ferris et al., 2008; Leung et al., 2011; Wu et al., 2012). Workplace ostracism also reduces job performance and productivity and increases self-defeating behaviors, such as procrastination (Leung et al., 2011; Renn et al., 2013). Additionally, ostracism is associated with increases in turnover intentions, or intentions to leave the workplace (Ferris et al., 2008).

Generally workplace ostracism has been studied as part of other mistreatment constructs and has only recently been recognized as a distinct construct from other forms of workplace mistreatment (Robinson et al., 2013). Other mistreatment behaviors, such as workplace bullying, sexual harassment, and incivility, involve a negative or unwanted behavior toward a target recipient, whereas workplace ostracism involves the withdrawal of interaction with the target worker (Ferris et al., 2008). As a result, workplace ostracism has a distinct impact on the targeted individual relative to other mistreatment behaviors. For instance, empirical studies found that workplace ostracism, compared to workplace bullying, has a stronger influence on work-related attitudes (e.g., affective commitment and psychological withdrawal) and job turnover (O’Reilly et al., 2014). Furthermore, the impact of workplace ostracism is greater than the effects of sexual harassment and incivility on emotional exhaustion, cynicism, and professional efficacy (Sulea et al., 2012).

A distinction can be made between the different forms that ostracism may take. Ferris et al.’s (2008) Workplace Ostracism Scale, for example, measures ostracism on a social level – situations where others have refused to engage the target in social interactions. This scale has been widely used in the workplace ostracism literature (e.g., Leung et al., 2011; Sulea et al., 2012; Wu et al., 2012). However, other forms of exclusion, such as information exclusion, should be examined. Information exclusion, or being ‘left out of the loop,’ occurs when targets are not physically or socially excluded from a group; rather, they are not privy to information that other group members share (Jones et al., 2009). Although considered more ambiguous and less powerful than social ostracism, being left out of the loop has negative effects on trust and liking of group members. Furthermore, the lack of information may reduce chances to receive some form of benefit (Jones et al., 2009). When information exclusion decreases the target’s ability to participate in a group task, then perceptions of participation affect the target individual’s perceptions of belonging, control, self-esteem, trust and liking of others, and their own competence (Jones et al., 2011).

## Workplace Ostracism and the Role of Gender

According to Kanter (1977), underrepresentation of a social group places the token members under increased scrutiny, and more balanced representation of social groups should relieve this scrutiny. For women who enter academia, there is evidence of both a ‘glass ceiling’ – underrepresentation at higher levels of the organization – and ‘glass walls’ – women being concentrated in particular fields or tracks. American Association of University Professors (2012) reported that 44% of women, compared to 62% of men, are tenured faculty and more female faculty (32.3%) are in non-tenure track positions, compared to male faculty (19%). Hence, female faculty hold a greater percentage of marginalized positions in academia, compared to the career paths of male faculty.

Acker (1990) suggests the gendered nature of organizations benefits men, who do not face societal expectations to care for family in the same manner as women. The inconsistency between women’s gender and academic work role is supported by research – women report greater academic stress, family stress, and less support of work/life balance compared to men (O’Laughlin and Bischoff, 2005). They may avoid academia due to perceived barriers related to parenthood (van Anders, 2004). In addition to these challenges, women may face interpersonal consequences based on their group membership.

According to social identity theory, people seek to maintain a positive and distinct social identity, in comparison to other groups (Turner and Reynolds, 2001). Threats to this identity – such as an increasing number of women in a male-dominated area – can lead to in-group bias and social competition (Turner and Reynolds, 2001). An outcome of this bias is the ‘chilly climate’ for women in academia – the perception of an exclusionary workplace environment. Indeed, women report greater exclusion from informal networks compared to male colleagues, affected by factors such as the perceived proportion of women to men within the department (Maranto and Griffin, 2011). Settles and O’Connor (2014) examined the experience of incivility – rude or discourteous behavior – not only within the workplace, but in another environment that can be important to networking and professional exposure – academic conferences. Women report more exclusion, incivility, and sexual harassment at conferences than men. Yet another study of university faculty found that more female faculty report gender harassment, verbal aggression, disrespectful behavior, and isolation/exclusion than male faculty (Richman et al., 1999). These subtle interpersonal interactions carry negative consequences for female faculty, as these experiences have negative effects on mental health and intentions to leave the workplace (Richman et al., 1999; Cortina et al., 2013).

Though research has established the effects of workplace ostracism (e.g., Hitlan et al., 2006a; Ferris et al., 2008; Zhao et al., 2013), little is known about its antecedents. Research from Milam et al. (2009) found that individuals who report experiencing frequent incidents of incivility are also identified by coworkers as provocative or “deserving” victims. Likewise, there may be characteristics that predispose certain people to being ostracized in the workplace. Social exchange theory provides a framework for understanding these characteristics.

The core of social exchange relationships is reciprocation (Rousseau and McLean Parks, 1993): social resources provided by a source individual or organization encourage dedication or reciprocation by the target receiver (Emerson, 1976; Cropanzano and Mitchell, 2005). These social resources can offer socioemotional outcomes including esteem and fulfillment of social needs. Social exchange theory has been used to explain a variety of interactional outcomes at work, including prosocial behavior toward coworkers, relationships between supervisors and subordinate employees, and organizational commitment (Cropanzano and Mitchell, 2005).

Given that perceived workplace ostracism experiences affect a majority of workers (O’Reilly et al., 2014), then social exchange theory can guide expectations as to who is more likely to experience this form of social exclusion. Researchers have found that targets of workplace ostracism are those who have displayed a tendency for uncooperative or disruptive workplace behaviors (Scott et al., 2013) or workers who prefer domination of coworkers to cooperation (Halevy et al., 2013). Conversely, more valuable partners report experiencing less ostracism despite their behavior toward others (Scott et al., 2013). These results parallel laboratory findings that show individuals may be ostracized as a means of social control against unhelpful group members (Wesselmann et al., 2013) or to conserve resources depleted by interaction with unpleasant individuals (Sommer and Yoon, 2013). Together, these studies show that those who are ostracized are often those who are not viewed as valuable exchange partners, either because they are unpleasant to interact with or because they have little to offer the designated group members in terms of resources.

However, studies conducted from the target’s perspective suggest another antecedent of workplace ostracism. Cortina et al.’s (2013) selective incivility theory proposes that uncivil workplace behaviors, such as rudeness or ostracism, occur as a modern form of discrimination, being especially likely to target women and ethnic minorities. As a modern form of discrimination, selective incivility is both subtle and ambiguous, allowing for perpetrators to justify their behavior as non-discriminatory (Cortina et al., 2013). This is congruent with another tenet of social exchange – status consistency (Meeker, 1971). Status consistency proposes that resources are distributed to others based on their status and social standing in the group, in order to maintain the status quo.

This effect may be especially prevalent in workplaces with changing demographics, such as academia. For example, Mehra et al. (1998) found that, for students in an MBA program, men’s tendency to form friendships with men (homophily) was related to greater centrality in a social network. For women, however, friendships with other women did not increase or decrease their centrality in social networks, suggesting that women’s friendships with other women were not the result of simply identification with other women, but the result of gender-based exclusion. The exclusion of women from men’s social networks, as seen in Mehra et al.’s (1998) study, maintains men’s social identities as positive and distinct from those of women. Threats to this identity – such as an increasing number of women in a male-dominated area – can lead to in-group bias and social competition (Turner and Reynolds, 2001).

Indeed, studies suggest that individuals belonging to stigmatized groups may experience workplace ostracism as a result of their collective group identity, rather than their actual workplace behaviors (O’Reilly and Robinson, 2009; Maranto and Griffin, 2011; Settles and O’Connor, 2014). For example, O’Reilly and Robinson (2009) found that employees attributed their experiences of workplace ostracism to group differences in age or race between coworkers. Research using measures (e.g., incivility and bullying) that include ostracism have shown that gender and ethnicity both play a role in predicting targets of workplace mistreatment (Cortina et al., 2001; Fox and Stallworth, 2005). This may indicate that workplace ostracism is not limited to those who display unwelcome behavior in the workplace, but is also affected by factors such as social identities.

Male-dominated workplaces may provide a context where gender may become especially salient, affecting social interactions. For example, women in academia – compared to men – perceive the workplace climate as more “chilly” or exclusionary, affected by factors such as the perceived proportion of women to men within the department (Maranto and Griffin, 2011). Women also report more social and intellectual exclusion at academic conferences compared to men (Settles and O’Connor, 2014). The present studies build upon this previous research by focusing on self-reported occurrences of two forms of ostracism behaviors – workplace ostracism and information exclusion. Drawing on these studies and guided by social exchange theory, we predict for the present study that women in academia will be more likely to experience workplace ostracism.

H1: Women in a male-dominated industry (academia) will report a greater number of workplace ostracism experiences (Study 1) and will report higher informational ostracism (Study 2) compared to men.

## Workplace Ostracism: Group Size vs. Group Status

Whereas women may be in the minority within the academic profession, they may not be in the minority within their specific university, college, or home department. This raises the question about whether one’s immediate environment is more or less important when it comes to feeling included. Theoretically, the status differential that leads to an unequal allocation of resources (i.e., social interactions) should decline as the ratio of men to women becomes more equal. Indeed, Maranto and Griffin (2011) find that university climate is perceived as more exclusionary when women’s perceived representation is low. We extend this research on representation by using an objective measure of gender representation, reducing the potential of relationship inflation due to common method bias. Therefore, we expect a given employee’s status as a numerical minority or majority within the department could override the negative effects of being in minority status within the overall organization.

H2: There will be a significant interaction between sex and proportional representation in the department on experiences of ostracism, such that women in departments with a high relative proportion of women will report less ostracism than women in departments with a low proportion of women.

## Study Overview

Participants were recruited at two time points to complete campus climate surveys that were distributed to department faculty members, via email in spring semesters 2013 and 2015, at a large, public, research university. These studies were carried out in compliance with ethical standards recommended in the Belmont Report and approved by the Texas A&M University Institutional Review Board. All participants gave informed consent according to the protocol required by the Texas A&M University Human Subjects Protection Program.

## Study 1 Method

### Participants and Procedure

Faculty participants were provided with a link to the web-based survey. In total, 1223 participants responded, a 45.46% response rate. The sample was comprised of 789 male faculty, 413 female faculty, and 4 transgender faculty from 100 departments (17 faculty chose not to disclose their sex), with a mean age of 50.69 years (*SD* = 11.79) and a mean organizational tenure of 13.25 years (*SD* = 10.64).

### Measures

#### Proportion of Women in Department

The proportion of women in each department was calculated using organizational records obtained from the Office of the Dean of Faculties. The number of women in each department was divided by the total number of faculty in each department. The resulting proportion was then matched to participant data using their self-reported department.

#### Workplace Ostracism

Workplace ostracism experiences were measured using an adaptation of the Workplace Ostracism scale (Ferris et al., 2008), modified to conserve space and follow the format of similar questions in the larger survey. Participants were asked, “During the past year, have you been in a situation in your department/unit where a (colleague/staff/student) …” followed by six descriptors of an ostracism situation (e.g., “Ignored you during conversation?”). As this was the first inclusion of the scale in a multi-wave climate survey, responses were truncated to Yes/No answers to establish occurrence without deviating from the format of the larger survey. Participants indicated either “Yes” or “No” to each descriptor, and, because of the low number of yes answers from staff/student sources, the total number of “Yes’s” for each source (colleague, staff, and student) was summed to create a frequency of workplace ostracism. Thus, scores could range from 0 to 18.

#### Rank

Faculty rank was measured by asking participants, “Please choose the title that best approximates your current job title:” followed by four options in increasing order of tenure, from non-tenure track faculty to tenured professor. Because junior rank in academia is associated with perceived stress (Thorsen, 1996), it was treated as a covariate in all analyses.

## Study 1 Results

Because participants were nested in departments, and the gender representation variable was assessed at the department level, we used multilevel modeling to test the hypotheses. The first step in the multilevel model analysis was estimating a base model with no level-one or level-two predictors, in order to model individual-level variance in workplace ostracism. Examination of the estimates for fixed effects indicate that, on average, employees reported 1.93 instances of ostracism (*SE* = 0.11). For this base model, the intraclass correlation (ICC) was estimated at 0.04, indicating that 4% of the variance in workplace ostracism experiences is due to department level characteristics. While the small ICC may suggest that a multilevel analysis is not necessary, there are benefits to using multilevel analysis even with small ICCs (Hayes, 2006); thus, we proceeded with the multilevel analysis. Stated as a final mixed model, the predicted equation is:

$$\begin{array}{l} \gamma_{ij}\,\;\;\;(workplace\,\;\;ostracism)=\gamma_{00}+\gamma_{10}\,\;\;(rank)+\gamma_{01}\,\;\;\left(gender\right)\\ \;\;\;\;\;\;\;\;\;\;\;\;\;\;\;\;\;\;\;\;\;\;+\gamma_{20}\,\;\;\left(department\,\;\;\;level\,\;\;\;gender\,\;\;\;representation\right)\\ \;\;\;\;\;\;\;\;\;\;\;\;\;\;\;\;\;\;\;\;\;\;+\gamma_{21}\,\;\;\left(department\,\;\;\;level\,\;\;\;gender\,\;\;\;representation*gender\right)\\ \;\;\;\;\;\;\;\;\;\;\;\;\;\;\;\;\;\;\;\;\;\;+u_{0j}+r_{ij} \end{array}$$

The model was estimated in three steps, beginning with a null model, and culminating with the simultaneous computation of the full model. These analyses are shown in **Table 1**. The column labeled Model 2 shows the level 1 predictor model, containing rank as a covariate and gender as a predictor. The column labeled Model 2 contains the interaction between gender and the level 2 variable, percentage of women in the department.

**Table 1:** Coefficients for multilevel models (Studies 1 and 2).

	Study 1		Study 2	
	Model 2	Model 3	Model 2	Model 3
Tenure	0.34^∗^	0.29^∗^	-0.02	-0.07
Gender	-0.46^∗^	-0.79	-0.01	0.19
Percent female in dept.		0.03		-1.23
Gender × Percent female		1.41		1.62

*p < 0.05

As the **Table 1** shows, there was a significant main effect for gender, γ_01_ = -0.46, *p* = 0.02. The negative sign on the coefficient indicates that women (coded as 0) reported significantly more ostracism than men (coded as 1), supporting Hypothesis 1. Contrary to Hypothesis 2, there was no significant interaction between gender and percentage of women in the department, γ_21_ = 1.41, *p* = 0.32.

## Study 1 Discussion

In this study, we examined the incidence of social ostracism in the workplace among male and female faculty members. We found that, consistent with our first hypothesis, female faculty reported greater incidence of workplace ostracism. However, the predicted interaction between percentage of women in the department and gender was non-significant; that is, we found no evidence that women in departments with a greater proportion of women experience less ostracism than women in departments with low numbers of women. In Study 2, we extend these findings to another form of ostracism – information exclusion. Furthermore, we replicate Study 1’s examination of workplace ostracism using a Likert-scale measure of ostracism frequency.

## Study 2 Method

### Participants and Procedure

Faculty participants were provided with a link to the web-based survey. In total, 1597 participants responded, a 35.85% response rate. The sample was comprised of 924 male faculty, 559 female faculty, and 6 transgender faculty from 100 departments (108 faculty chose not to disclose their sex), with a mean age of 50.42 years (*SD* = 12.00) and a mean organizational tenure of 12.67 years (*SD* = 11.05).

### Measures

#### Proportion of Women in Department

The proportion of women in each department was calculated using organizational records obtained from the Office of the Dean of Faculties. The number of women in each department was divided by the total number of faculty in each department. The resulting proportion was then matched to participant data using their self-reported department.

#### Information Exclusion

Experiences of information exclusion were measured using an eight item scale (Jones et al., 2009). Participants were asked, “How frequently do you feel or experience these things at work? Please respond as accurately and honestly as possible…” followed by six statements of informational inclusion or exclusion (e.g., “Through no fault of my own, I seem to be one of the last to find out about information at work.” Participants indicated the frequency of these experiences, from 1 – Never or almost never to 5 – Almost always. Items were reverse coded as needed and averaged to create a measure of information exclusion.

#### Workplace Ostracism

Workplace ostracism experiences were measured using a nine item scale. Source information was not collected in Study 2; however, due to changes in the length and format of the larger survey, we were able to include Likert scale measures of frequency. Participants indicated the frequency of these experiences, from 1 – Never or almost never to 5 – Almost always. Items were reverse coded as needed and averaged to create a measure of workplace ostracism.

#### Rank

Faculty rank was measured by asking participants, “Please choose the title that best approximates your current job title:” followed by four options in increasing order of tenure, from non-tenure track faculty to tenured professor. As in Study 1, rank was treated as a covariate in all analyses.

## Study 2 Results

### Information Exclusion

The same process for building the multilevel model was used from Study 1. First, we estimated a base model with no level-one or level-two predictors, in order to model individual-level variance in information exclusion. Examination of the estimates for fixed effects indicate that, on average, employees reported a mean frequency of 2.90 on information exclusion (*SE* = 0.02) – for reference, a 3 was labeled as “Sometimes.” For this base model, the ICC was estimated at 0.08.

Descriptive information and correlations for Study 2 variables are seen in **Table 1**. The predicted equation for the full mixed model is:

$$\begin{array}{l} \gamma_{ij}\,\;\;\;(information\,\;\;exclusion)=\gamma_{00}+\gamma_{10}\,\;\;(rank)+\gamma_{01}\,\;\;\left(gender\right)\\ \;\;\;\;\;\;\;\;\;\;\;\;\;\;\;\;\;\;\;\;\;\;+\gamma_{20}\,\;\;\left(department\,\;\;\;level\,\;\;\;gender\,\;\;\;representation\right)\\ \;\;\;\;\;\;\;\;\;\;\;\;\;\;\;\;\;\;\;\;\;\;+\gamma_{21}\,\;\;\left(department\,\;\;\;level\,\;\;\;gender\,\;\;\;representation*gender\right)\\ \;\;\;\;\;\;\;\;\;\;\;\;\;\;\;\;\;\;\;\;\;\;+u_{0j}+r_{ij} \end{array}$$

These analyses of Models 2 and 3 are shown in **Table 1**. As the table shows, there was a no effect for gender, γ_01_ = -0.01, *p* = 0.89, contrary to Hypothesis 1. Similarly, there was no significant interaction between gender and percentage of women in the department, γ_11_ = 1.62, *p* = 0.14.

### Workplace Ostracism

We used the same process from Study 1 for building the multilevel model for workplace ostracism. Examination of the estimates for fixed effects indicate that, on average, employees reported a mean frequency of 1.33 on workplace ostracism (*SE* = 0.02) – for reference, a 3 was labeled as “Sometimes.” For this base model, the ICC was estimated at 0.07.

Descriptive information and correlations are seen in **Table 1**. There was a significant effect for gender, γ_01_ = -0.15, *p* < 0.001. The negative coefficient indicates that women (coded 1) experienced a greater frequency of workplace ostracism than men (coded 0), supporting Hypothesis 1. Similar to Study 1, there was no significant interaction between gender and percentage of women in the department, γ_11_ = 0.05, *p* = 0.84.

### Additional Analyses

Unlike Study 1, we had a sufficient number of respondents who indicated their ethnicity for us to include it as a variable. Previous research on workplace ostracism has indicated that differences in ethnicity may contribute to ostracism experiences (O’Reilly and Robinson, 2009). Furthermore, research on Faculty of Color in academia found that faculty men and women of color feel more isolated than White male or female faculty (Smith et al., 2005). Based on this, we conducted additional analyses assessing the interaction of ethnicity and gender on the experience of both social ostracism and information exclusion. Because of the large discrepancies in number between White Faculty and Faculty of Color, we condensed responses into a binary variable indicating White or Person of Color.

Results for the model predicting ostracism were similar to the results of Study 1. There was a significant main effect for gender, γ_01_ = -0.11, *p* = 0.01. There was no significant main effect for ethnicity, γ_20_ = 0.09, *p* = 0.20, nor was there a significant interaction between ethnicity and gender, γ_21_ = -0.03, *p* = 0.76.

For the model predicting information exclusion, there was a significant main effect of ethnicity (γ_20_ = 0.34, *p* < 0.001), such that Faculty of Color reported more frequent information exclusion than White Faculty. No significant effect for gender (γ_01_ = 0.06, *p* = 0.27) or interaction between gender and ethnicity were seen (γ_21_ = -0.16, *p* = 0.15).

## General Discussion

The goal of this study was to identify gender differences in both social and informational ostracism experiences among women in academia. Based on previous research (e.g., Maranto and Griffin, 2011), we predicted that female faculty would experience more workplace ostracism and information exclusion than male faculty. Our hypothesis was partially supported: across two studies, women had greater perceived incidence of workplace ostracism compared to men. This finding supports existing research on the “chilly” climate in academia that creates an exclusionary environment for women, both at the workplace and at work-related events (Maranto and Griffin, 2011; Settles and O’Connor, 2014). However, contrary to hypothesis, women did not report a greater frequency of information exclusion in Study 2.

The proportion of women within the department did not have a significant effect on the amount of workplace or informational ostracism experienced; women were equally likely to be ostracized in male-dominant departments as were women in departments with more equal proportions. The discrepancy between our findings and the results of Maranto and Griffin (2011) may be due to methodological differences: Maranto and Griffin (2011) used a subjective measure of the proportion of women in department (i.e., self-reported perceptions of departmental composition), whereas the present study used an objective measure of the proportion of women in department from organizational records. If the differences in findings are due to the measurement, this suggests that it is not so much the number of women in the department that matters as much as the number of women recalled or salient to the employee which may be based on physical presence and/or amount of interaction with the employee.

Another potential explanation is in the differences of measuring exclusion and ostracism. For example, Maranto and Griffin’s exclusion measure used items that included not only social situations, e.g., “I feel welcome and included in informal and social gatherings…” but also information exclusion (e.g., In my department, colleagues do not share some job-related information with me…” and perceptions of social hierarchy (e.g., “An old boy’s network runs my department”). Therefore, the effect of department gender composition found in their study may reflect a reduction in perceived exclusion in a more general definition than that of ostracism, which specifically examines occurrences of being ignored or excluded by others.

The lack of a gender difference for information exclusion is surprising, given that the more blatant form of exclusion, workplace ostracism, was more frequent for female faculty. However, previous research by Smith et al. (2005) has shown that feelings of social and institutional isolation differ based on gender. They found that female faculty had greater perceptions of exclusion from supportive networks; however, male and female faculty were similar in their perceptions of access to organizational resources. The present studies mirror these findings, in that women report greater ostracism in social, rather than informational, interactions.

The additional analyses of Study 2 provide further insight into the experiences of underrepresented groups in academia. While there was no difference in information exclusion comparing men and women, we did find that Faculty of Color reported greater frequency of exclusion than White faculty. However, there was no difference between White faculty and Faculty of Color in social ostracism in the workplace. This suggests that while underrepresented groups – both in terms of gender and ethnicity – may experience ostracism, the form it takes differs. This may indicate particular difficulties for faculty women of color, who, due to their intersectional identities, may experience higher levels of both social and informational ostracism in the workplace.

### Theoretical and Practical Implications

An outcome of this research is that it can help identify who may be most vulnerable to experiencing workplace ostracism. This identification provides an alternative view to the current literature on antecedents of ostracism, which places responsibility solely on the target for being uncooperative, unhelpful, or unfriendly (e.g., Halevy et al., 2013; Scott et al., 2013; Wesselmann et al., 2013). While social exchange theory explains the ostracism of low-value exchange partners (e.g., those who are uncooperative or burdensome), the present research extends the application of social exchange theory from the rule of reciprocity to the status consistency rule (Meeker, 1971). This supports the use of social exchange principles in explaining workplace phenomenon and provides new directions for research using the status consistency framework.

Due to the nature of this sample, the results of this study may provide insight into the unique experiences of women in a traditionally male-dominated environment. Because the proportion of women did not influence the chances of experiencing ostracism, this suggests that social status processes and broader issues of institutional/departmental climate may be key in determining who may be at risk; this information may be of particular use to organizations who are concerned with increasing and retaining employees from diverse populations, especially those from groups which are culturally stereotyped as being of lower status. Another contribution of this study to understanding women’s experiences in academia is the presence of source information in Study 1. Overwhelmingly, the source of workplace ostracism was other faculty members, rather than staff or students. Awareness of the occurrence and risk factors for ostracism in the workplace may help improve the effectiveness of diversity initiatives.

### Strengths, Limitations, and Future Directions

A strength of the present design was that we collected data from two sources rather than a single source, which reduce concerns related to single-source bias (Podsakoff et al., 2003). Specifically, the variable “proportion of women in department” was collected from organizational records obtained from the Office of the Dean of Faculties, whereas the other variables, such as workplace ostracism and faculty rank were collected from self-report surveys. Although it is argued that the majority of variables that are collected via self-report survey might inflate the observed relationships, Conway and Lance (2010, p. 327) theorized that “the same-method observed score correlations are actually quite accurate representations of their true-score counterparts,” and self-reports are not inferior to other-reports or other methods. Actually, self-reports are clearly appropriate for constructs that consist of personal or private feelings and thoughts like workplace ostracism and information exclusion (Chan, 2009).

However, we acknowledge some limitations of the present design. That said, the cross-sectional data did not allow for causal inference. However, our hypotheses were guided by relevant theories and empirical studies. To establish causal relationships, other designs (e.g., experimental design or longitudinal studies) are needed to replicate the present findings.

In terms of generalizability, this study is limited in that it consists of data collected from single samples in a work environment that research has established as being particularly exclusionary toward women (e.g., Maranto and Griffin, 2011). Workplaces that are historically more inclusive of women, or even female-dominated, may not have the same risk of workplace ostracism for women.

Future studies should more closely examine ethnicity as a potential antecedent of workplace ostracism. Findings from Welbourne et al. (2014) indicated that employees’ ethnicity and cultural values may predict their vulnerability to the impact of incivility at work. Similarly, studies show that employees from underrepresented ethnic groups may be at particular risk for experiencing workplace ostracism (Turner et al., 2008; O’Reilly and Robinson, 2009). Detailed comments in our data from participants support the need to examine the experiences of workers from underrepresented ethnic groups (e.g., “I have heard ‘coded’ racist remarks in my department…,” and “Racism! Specifically toward black men, from both faculty and students.”) Moreover, future research should consider ethnicity and race-based stereotypes – especially in White-dominated workplaces – to support the status consistency determinant of workplace ostracism.

The lack of a significant effect for the proportion of women in the department may suggest that it is not as simple as an issue of representation. Research on men in female-dominated occupations illustrates the importance of gendered stereotypes in determining when lower representation leads to negative outcomes (i.e., the glass ceiling or sticky floor effect) or positive outcomes (i.e., the glass escalator effect; Budig, 2002). However, the low variance in the proportion of women (*SD* = 0.17 in Study 1 and *SD* = 0.18 in Study 2) may also have contributed to this null finding. In both studies, the vast majority of participants (83.1% in Study 1; 80.2% in Study 2) were in departments with more male than female faculty. A third factor that may have influenced the lack of effect for gender representation is the effects of broader climate issues that contribute to perceptions of exclusion; Maranto and Griffin (2011) found that perceptions of procedural justice and gender equity had a large impact on perceived exclusion. Future research should examine if these climate factors affect ostracism behaviors perceived by targets from underrepresented groups in academia.

## Conclusion

We found that female faculty experienced greater amounts of workplace ostracism than male faculty, regardless of the departmental proportion of women. Additionally, we found that experiences of a more subtle form of ostracism, information exclusion, was greater among Faculty of Color as compared to White faculty. These results support Cortina et al.’s (2013) assertion that workplace mistreatment occurs as a subtle form of discrimination. While workplace policies that prohibit sexual harassment, bullying, and other forms of explicit mistreatment on the basis of gender exist, workplace ostracism can be subtle, ambiguous, or even unintentional, causing it to be more difficult to police than mistreatment that involves interactions toward an individual (as opposed to a lack of interaction). However, the negative consequences of workplace ostracism suggest that it should be of interest to organizations, particularly if they are concerned with aiding the psychological health and retention of underrepresented groups through support interventions.

## Author Contributions

CZ made substantial contributions to the conception and design of the work, plus the acquisition, analysis, and interpretation of data for the work. CZ drafted the work for critically important intellectual content. CZ will serve as first author on the submission. AC-S made substantial contributions to the conception and design of the work, plus the interpretation of data for the work. AC-S also revised the final document for critically important intellectual content and has final approval of the version to be published. AC-S agrees to be accountable for all aspects of the work in ensuring that questions related to the accuracy or integrity of any part of the work are appropriately investigated and resolved. AC-S will serve as both corresponding and second author on the submission. XX made substantial contributions to the conception and design of the work, plus the acquisition, analysis, and interpretation of data for the work. XX edited the work for critically important intellectual content. XX will serve as third author on the submission.

## Conflict of Interest Statement

The authors declare that the research was conducted in the absence of any commercial or financial relationships that could be construed as a potential conflict of interest.
